# Retrospective cross-validation of automated sleep staging using electroocular recording in patients with and without sleep disordered breathing

**DOI:** 10.1186/1755-7682-5-21

**Published:** 2012-06-25

**Authors:** Daniel J Levendowski, Djordje Popovic, Chris Berka, Philip R Westbrook

**Affiliations:** 1 Advanced Brain Monitoring, Inc, 2237 Faraday Avenue, Carlsbad, CA 92008, USA

## Abstract

**Background:**

Alterations of sleep duration and architecture have been associated with increased morbidity and mortality, and specifically linked to chronic cardiovascular disease and psychiatric disorders, such as type 2 diabetes or depression. Measurement of sleep quality to assist in the diagnosis or treatment of these diseases is not routinely performed due to the complexity and cost of conventional methods. The objective of this study is to cross-validate the accuracy of an automated algorithm that stages sleep from the EEG signal acquired with sensors that can be self-applied by patients.

**Methods:**

This retrospective study design included polymsomnographic records from 19 presumably healthy individuals and 68 patients suspected of having sleep disordered breathing (SDB). Epoch-by-epoch comparisons were made between manual vs. automated sleeps staging (from the left and right electrooculogram) with the impact of SDB severity considered.

**Results:**

Both scoring methods reported decreased Stage N3 and REM and increased wake and N1 as SDB severity increased. Inter-class correlations and Kappa coefficients were strong across all stages except N1. Agreements across all epochs for subjects with normal and patients with mild SDB were: wake = 80%, N1 = 25%, N2 = 78%, N3 = 84% and REM = 75%. Agreement decreased in patients with moderate and severe SDB amounting to: wake = 71%, N1 = 30%, N2 = 71%, N3 = 65%, and REM = 67%. Differences in detection of sleep onset were within three-minutes in 48 % of the subjects and 10-min in 73 % of the cases and were not impacted by SDB severity. Automated staging slightly underestimated total sleep time but this difference had a limited impact on the respiratory disturbance indexes.

**Conclusions:**

This cross-validation study demonstrated that measurement of sleep architecture obtained from a single-channel of forehead EEG can be equivalent to between-rater agreement using conventional manual scoring. The accuracies obtained with automated sleep staging were inversely proportional to SDB severity at a rate similar to manual scorers. These results suggest that the automated sleep staging used in this study may prove useful in evaluating sleep quality in patients with chronic diseases.

## Background

Adequate amounts and quality of sleep are essential for health and well-being. Both short and long sleep durations are significant predictors of morbidity and all-cause mortality [1]. Short duration sleep (< 6 hours per night) represents an independent risk factor for development of type 2 diabetes [2-7], central obesity (in women) [8] and psychiatric disorders such as depression, attention deficit and substance abuse [9-14]. A lack of certain phases of sleep may have adverse effects on health in spite of the seemingly adequate sleep duration. For example, an insufficient amount of slow wave sleep has been associated with hypertension [15], type 2 diabetes [3,16] and increased risk of obesity [17], while anomalies of rapid eye movement (REM) sleep have been linked to dementia, depression and post-traumatic stress disorder (PTSD) [18-20].

Conventionally, the assessment of sleep architecture has been done in dedicated facilities and relied on multichannel polysomnography (PSG) and manual scoring of the data. PSG provides comprehensive information about sleep duration and architecture but it is too expensive and cumbersome for large-scale or repeated-measures evaluations. Manual scoring is a laborious practice even with the use of software to assist with scorers [21], and furthermore it is subject to considerable disagreement between experts in regard to assigned sleep stages, cumulative measures of sleep structure and indices of respiratory or other disturbances [22-24]. On the other hand, inexpensive tools that are validated and applicable on a large scale (i.e., wrist actigraphy and sleep diaries) provide only rudimentary estimates of total sleep time without any information about the quality of periods self-reported or labeled by actigraphy as sleep. As a result, sleep is rarely evaluated in patients with suspect or confirmed chronic disorders where its assessment might provide important clues for diagnosis or treatment.

Recent advances in electronic technologies and sensor interfaces have allowed for a significant reduction of the size and weight of recording equipment and made its self-application feasible. This would allow assessment of sleep quality in the home where a patient’s sleep patterns can be objectively quantified in their normal sleeping environment using wearable recorders with only few EEG electrodes below the hairline. A compelling advantage of such an approach is that adhesive ECG-type electrodes or pads made from conductive fabrics can be easily applied to the forehead or around the eyes by patients following a simple set of instructions. In recent years several algorithms for automated staging of sleep from a limited number of channels have been introduced and successfully validated in samples composed mostly or exclusively of healthy volunteers [25-27]. However similar levels of scoring accuracy need to be demonstrated in relevant clinical populations in order to support routine adoption.

This paper presents validation of a novel algorithm that stages sleep in conventional 30-second epochs from a single EEG channel. The algorithm was developed and initially validated in healthy subjects using a differential recording from two forehead electrodes (Fp1-Fp2) [28]. In this study the validation has been extended to a clinical population composed mostly of subjects evaluated with laboratory PSG for suspected sleep-disordered breathing. As the frontopolar electrodes are not routinely applied during such evaluations, the differential input for the algorithm was derived from the left and right electrooculographic (EOG) channels.

## Methods

### Subjects

Eighty-seven nocturnal polysomnographic records selected for this study were acquired under IRB review at the New York University Sleep Disorders Center. Prior to further stratification by sleep disordered breathing severity, the data set included 19 subjects presumed to be healthy and the balance suspected of having sleep disordered breathing (SDB). The ethnic profile included 52% white, 10% Asian, 9% African American, 9% Hispanic/Latino, and 20% unreported.

### Manual scoring

The montage used for manual sleep staging provided electro-encephalographic recordings from C3, C4, O1, O2 and Fz (referenced to the linked mastoids), left and right electrooculography (LEOG and REOG) and submental electromyography (EMG). One of several sleep technicians working in the laboratory during a one-year period beginning in March 2005 manually scoring each study using the criteria developed by [Rechtschaffen and Kales (R&K) 29] as incorporated into their clinical scoring protocols. The Apnea/Hypopnea Index (AHI) was based on 10-second cessation in breathing or a 30% reduction in airflow coupled to a 4% decrease in oxyhemoglobin saturation. The Respiratory Disturbance Index (RDI) was based on the Chicago research criteria [30]. Subjects were stratified into four SDB severity categories: normal (RDI <10), mild (RDI 10 – 20), moderate (RDI 21 – 40) or severe (RDI > 40) sleep disordered breathing (Table 1).

**Table 1:** Distribution of group data based on sleep disordered breathing severity

	**Normal**	**Mild**	**Moderate**	**Severe**
n (% male)	24 (42)	21 (86)	18 (83)	24 (83)
Age, mean yrs ± SD	39 ± 14.6	41 ± 11.4	53 ± 15.8	47 ± 12.1
AHI, mean ± SD	1 ± 1.3	4 ± 2.7	14 ± 8.7	57 ± 24.4
RDI, mean ± SD	6 ± 2.3	15 ± 3.1	31 ± 6.7	71 ± 19.3

### Automated scoring

**Figure 1:**
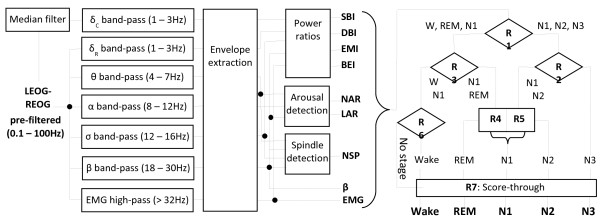
**Block diagram of the algorithm for automated sleep staging.** SBI – ratio of the average sigma and beta power; DBI – ratio of delta and beta power; BEI – ratio of beta and EMG power; EMI – ratio of the delta power before and after median filtering; NAR, LAR – number and length of arousals; NSP – number of sleep spindles.

Automated sleep staging was performed by the algorithm previously described [28] using a differential EEG recording (Fp1- Fp1) and validated against the 2007 AASM scoring rules [31] in rested and sleep deprived healthy individuals. The algorithm (Figure 1) first computes power estimates in the standard EEG frequency bands (delta, theta, alpha, sigma, beta) and extracts the eye movements, EMG, arousals and sleep spindles, and then feeds the power estimates, their ratios and the number of spindles and arousals in the epoch to a hierarchical decision tree that classifies each 30-second epoch into one of the five stages: Wake, REM sleep, NREM stage N1 (N1), NREM stage 2 (N2) and NREM stage 3 (N3 or slow-wave sleep).

Minor modifications have been made to the algorithm prior to its application to this study’s data. As the frontopolar differential EEG derivation was not available in this study, the algorithm was applied to the differential signal derived by subtracting the left EOG from the right EOG channel. As the filters in the sleep staging algorithms are designed for a sampling rate of 256 Hz, the difference EOG signal, originally available at 128 Hz, was subsequently re-sampled at 256 Hz using linear interpolation. Finally, the EMG power threshold was reduced by two thirds and the BEI threshold was scaled up by a factor of three in order to reflect the reduced width of the effective EMG band available in this study (32–64 Hz as compared to 32–128 Hz in the original data set [28]). The validity of these modifications was verified by an ad-hoc analysis of 19 records from the healthy subjects and 18 studies with manually staged total sleep time less than 3.5 hours. It is important, however, to stress that the vast majority of the algorithm’s thresholds (21 out of 23) were not changed.

### Analyses

Inter-class correlations were used to assess concordance across subjects between the total time spent in each of the stages as determined by manual vs. auto-scoring. Cross-tabulations were made between the manual and automated scoring for all epochs, and the overall and stage-specific percentage agreement and Kappa coefficients were calculated. Based on similarities in the scoring accuracy, the data from the subjects with normal and patients with mild SDB data were pooled into one and the data from patients with moderate and severe SDB into a second group. The variability of stage-specific agreement across subjects was analyzed on box-whisker plots for each of the five AASM sleep stages. Bland-Altman plots were used to assess differences between manual and automated estimates of total sleep time, sleep efficiency (ratio of the total sleep time and total recording time), and respiratory disturbance index (RDI) across all subjects. Sleep onset was identified from both manual and automated scoring (as three consecutive non-wake epochs) and the absolute differences between the two estimates were analyzed as a function of SDB severity and accuracy of detection of stages Wake and N1 in each particular subject.

## Results

### Correlations across staged time

The inter-class correlations across subjects with respect to minutes staged by manual and auto-scoring is presented in Table 2. The strongest concordance was in stage N3 and the weakest in Stage N1.

**Table 2:** Inter-Class Correlation (ICC) between manual and auto-scored time by stage

	**ICC**	**p <**
Wake	0.58	0.0001
N1	0.37	0.001
N2	0.76	0.0001
N3	0.87	0.0001
REM	0.75	0.0001

### Pooled accuracy by stage

Table 3 shows the agreements between the manual (M) and automated (A) scoring; each row representing the stage assigned by manual scoring and each column representing the stage assigned by the automated algorithms. With the exception of stage N1, the stage-specific agreement as measured with the respective Kappa coefficients was substantial in the group of healthy individuals and patients with mild SDB, and moderate in patients with moderate and severe SDB. For epochs manually staged as Wake, misclassifications were distributed fairly evenly between N1 and N2 (9.2 and 7.7% respectively). For manually staged N1, the preponderance of misclassified epochs were assigned to stage N2 (38.4%) followed by Wake (24.7%) and REM (11.2%). The misclassifications between N3 and N2 (13.5%) and REM and N2 (12.4%) occurred mostly in borderline epochs, i.e., during transitions between these stages.

**Table 3:** Pair-wise epoch by epoch agreement by SDB group

	**Automated staging**							
	Normal and Mild SDB (n = 40,641 epochs)							%
	Wake	N1	N2	N3	REM	Total	No. epochs	Epochs
Wake	79.7%	9.2%	7.7%	0.8%	2.6%	100%	7342	18.1%
NREM1	24.7%	25.1%	38.4%	0.6%	11.2%	100%	5436	13.4%
NREM2	6.2%	7.6%	77.7%	6.0%	2.4%	100%	18167	44.7%
NREM3	1.8%	0.0%	13.5%	83.9%	0.9%	100%	4234	10.4%
REM	4.9%	6.6%	12.4%	1.4%	74.6%	100%	5462	13.4%
No. epochs	8663	3793	18019	4817	5349			
% epochs	21.3%	9.3%	44.3%	11.9%	13.2%			
Kappa	0.67	0.21	0.60	0.76	0.72			
Moderate and Severe SDB (n = 29,938 epochs)								
	Wake	N1	N2	N3	REM	Total		
Wake	71.1%	16.9%	8.1%	0.3%	3.6%	100%	9086	22.4%
NREM1	18.9%	30.0%	40.3%	0.4%	10.5%	100%	6955	17.1%
NREM2	8.6%	14.1%	70.5%	4.1%	2.7%	100%	10205	25.1%
NREM3	7.1%	0.0%	26.7%	65.0%	1.1%	100%	1077	2.7%
REM	4.5%	10.8%	15.9%	1.4%	67.4%	100%	2615	6.4%
No. epochs	8851	5349	11430	1204	3104			
% epochs	29.6%	17.9%	38.2%	4.0%	10.4%			
Kappa	0.60	0.17	0.48	0.60	0.58			

Based on manual scoring, the distribution of sleep architecture changed across the two groups as SDB severity increased, most noticeable with stage N2 (44.7 vs. 25.1%), stage N3 (10.4 vs. 2.7%) and REM (13.4 vs. 6.4%). There were also important differences in the agreement between manual and automated staging when the normal and mild SDB group was compared to the moderate and severe SDB group. The percentage of epochs identified as Wake by visual scoring and staged as N1 increased from 9.2% to 16.9% due to the influence of obstructive breathing on sleep continuity. Similarly, the percentage of epochs visually scored as N2 and staged as N1 increased across the two groups from 7.6% to 14.1%. The concordance for N3 decreased from 83.9% to 65.0%, with misclassifications shifting to N2 likely as a result of a decreased amount and continuity of slow wave sleep in patients with more severe SDB. Finally, the increase in REM sleep misclassified as N1 (10.8% vs. 6.6%) for those with moderate/severe SDB was likely influenced by the overall reduction in REM time.

Kappa coefficients showed strong agreement for Wake, N3 and REM and moderate agreement for N2 in the normal and mild SDB group. In patients with moderate and severe SDB, Kappa coefficients showed moderate agreement for Wake, N2, N3 and REM.

### Staging accuracy by subject

**Figure 2:**
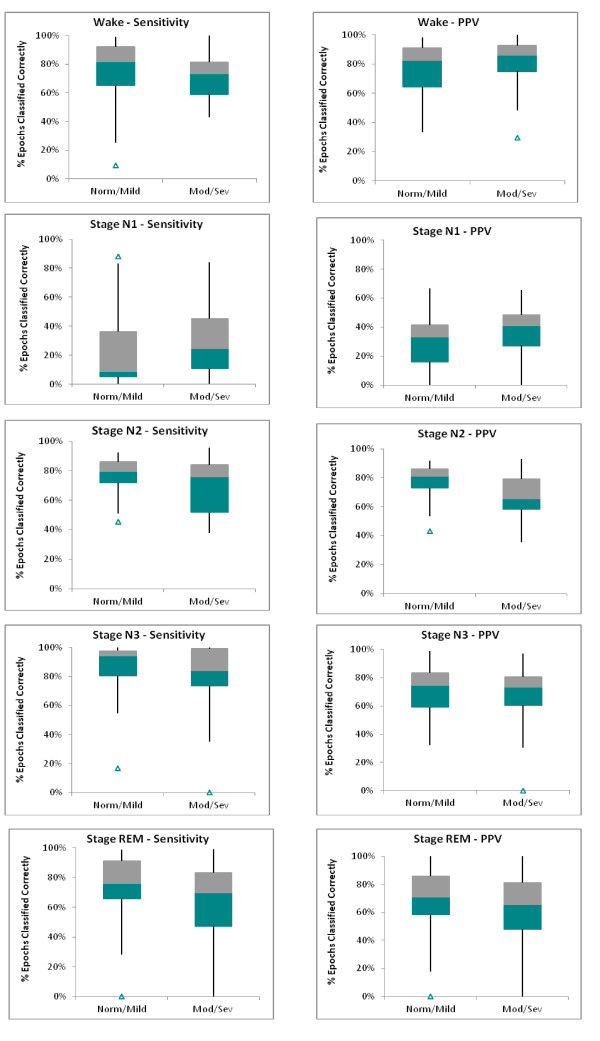
**Box-Whisker plots comparing stage-specific sensitivity and positive predictive value (PPV) by SDB severity.** The box represents the distributions of the 2nd and 3rd quartile about the median, the whiskers represent the 10% and 90%, and the Δ identifies outliers. Only subjects with a minimum of 20 manually scored epochs of the pertinent stage were included the respective plot.

The box-whisker plots in Figure 2 present the distributions of the stage-specific sensitivity and positive predictive value (PPV) across individuals and SDB severity. The two halves of the box represent than range of accuracies for the quartiles above and below the median value for the SDB group. The median sensitivity for Wake was 80% for the normal/mild and 70% for the moderate/severe SDB groups. The median distributions for all of the stages were this range other than Stage N1. Variability in the individual accuracies about the median (i.e., length of the box and whiskers) were fairly high for both sensitivity and PPV across all stages and SDB groups, and, with the exception of stage N2, the distributions were not markedly affected by SDB severity. Both sensitivity and PPV distributions for stage N2 significantly widened in patients with moderate and severe SDB as a result of an increased confusion of this stage with stage N1 (see also Table 3). The improved PPV for wake in patients with moderate/severe SDB is likely the result of rapid onset of sleep after lights out.

### TST and sleep efficiency

**Figure 3:**
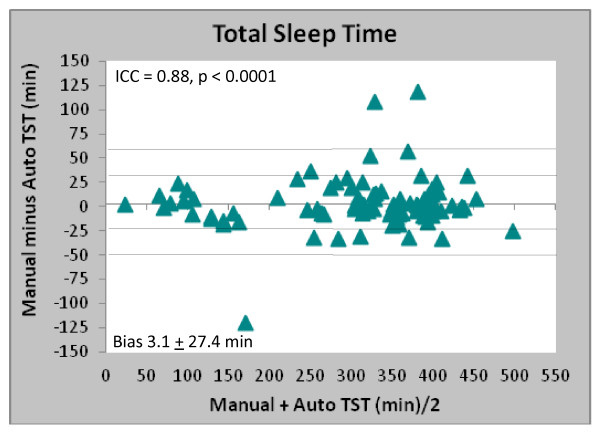
Bland-Altman plot comparing estimates of total sleep time (TST) for manual vs. automated scoring.

The Bland-Altman plot in Figure 3 highlight the differences in the measured total sleep time. There was a slight bias toward under-reporting the total sleep time (TST) and the maximum expected error in TST would be ± 17.4% (based on the SD of 27.4 min x 2 and mean TST of 310).

**Figure 4:**
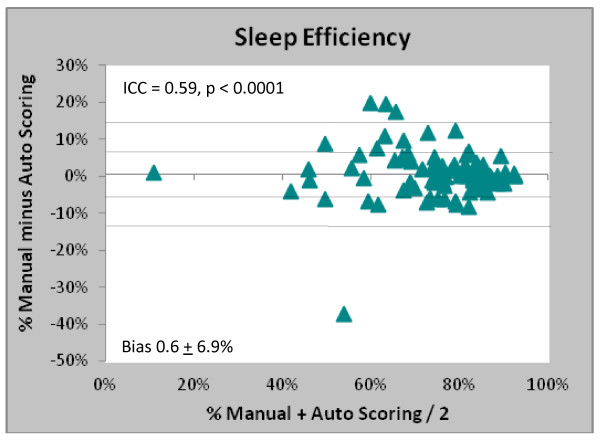
Bland-Altman plot comparing estimates of sleep efficiency for manual vs. automated scoring.

The Bland-Altman plot in Figure 4 shows there was no bias in the measurement of sleep efficiency when the automated scoring is compared to manual scoring, with sleep efficiency under-reported by a maximum of 13.8% for patients two standard deviations from the mean.

**Figure 5:**
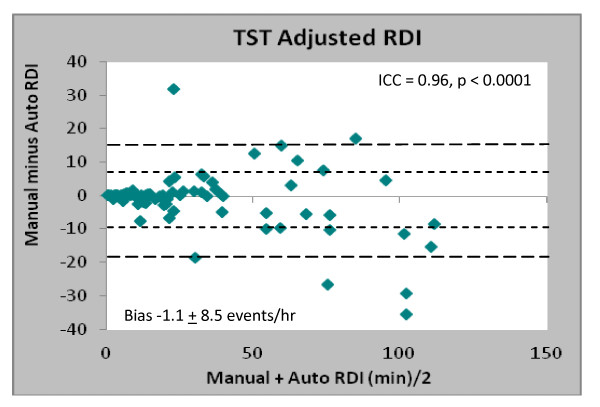
Bland-Altman plot comparing the RDI calculated from total sleep time (TST) derived from manual vs. automated sleep staging.

The impact of differences in total sleep time between manual and auto-scoring on the computed respiratory disturbance index is presented in Figure 5. Of the 13 cases that had an RDI difference greater than 10 events/hour, eight were severe, and the differences would not have affected the diagnoses.

### Sleep onset

The effect of Wake/sleep misclassifications was most obvious in the automated detection of sleep onset, where the absolute difference between the manual and automatic estimate was notable (i.e., > 3 minutes) in over 50% of the studies, with differences of over 10 minutes occurring in 27% of the subjects (Table 4). The error in the sleep onset detection did not seem to be related to the SDB severity. Automated scoring tended to underestimate sleep latencies: sleep onset was detected earlier by the algorithm in 73%, 90% and 84.6% of the subjects in the 4 – 10, 11 – 20 and > 20 minute groups respectively.

**Table 4:** Distribution of subjects by SDB severity and sleep onset difference categories

**n (%)**	**≤ 3 min**	**4 to 10 min**	**11 to 20 min**	**> 20 min**	**Total**
Normal/Mild	22 (49)	12 (27)	7 (15)	4 (9)	45
Moderate/Severe	20 (48)	10 (24)	3 (7)	9 (21)	42
Total	42 (48)	22 (25)	10 (12)	13 (15)	87

**Figure 6:**
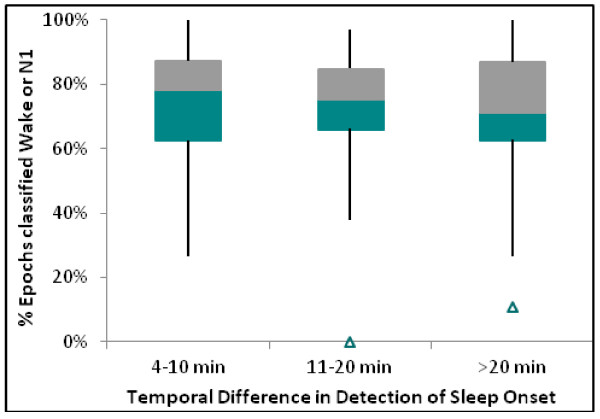
Box-Whisker plots across all subjects showing the percentage of epochs classified as Wake or N1 by automated (manual) scoring subsequent to its detection of sleep onset but prior to recognition of sleep onset by manual (automated) scoring.

Given the automated scoring identified sleep onset first in most of the cases where there was substantial disagreement, it was important to determine if the misclassifications resulted from brief periods of stage N1 followed by either wake or stage N1 (which is difficult to score by either method) or the epochs were staged as deeper sleep. Figure 6 presents a box-whisker plot in which the percentage of epochs stages as either wake or N1 subsequent to the detection of sleep onset by one method but before the detection by the other method. These results indicate the in a majority of the cases, 60 – 85% of the epochs were classified as wake or very light sleep during the periods where there was disagreement in the detection of sleep onset.

## Discussion

This study evaluated the accuracy of a novel algorithm designed to stage sleep from a single EEG channel using two sensors on the forehead. Overall, the agreement between the manual and automated scoring for all stages decreased as the severity of SBD increased, but remained fairly high for stages Wake, N2 and REM. The agreement by stage across the normal/mild SDB group were remarkably similar. For the moderate/severe SDB group the overall agreement dropped as the algorithm tended to classify into ‘lighter’ stages as compared to the human scorer, i.e. scored N1 in place of stages N2, REM epochs as N1 or N2, and N2 in place of manually scored N3. The agreement between manual and automated scoring for stage N1 was generally poor irrespective of the SDB severity, and this stage was typically confused with Wake or N2. While the disagreement between stages N1 and N2 is not relevant for most clinical situations (where the two stages are often viewed as a single ‘light NREM’ stage), unbalanced misclassification of a large proportion of N1 epochs as Wake or vice versa could have an impact on total sleep time and RDI estimates.

The Bland-Altman plot of manual and automated estimates of total sleep time suggest the misclassifications of N1 as Wake were, on average, counterbalanced by misclassification of Wake as sleep, as the bias for TST estimates was negligible and variance clinically insignificant. The wake/sleep misclassifications were stronger as the SDB severity increased, in part because there were more wake epochs. The influence of the Wake/sleep misclassification on the automated estimates of RDI was, nonetheless, negligible.

The validity of an algorithm for automated sleep staging is often assessed by comparison of its overall and stage-specific accuracy to the agreement between two (or more) human raters who scored the same data. This study was limited by the fact that we compared accuracy to one set of sleep scores per patient, with several technicians contributing to the scoring of the data set. The approach reduced the bias that may have been introduced by a single rater, or the introduction of “invalid” epochs if two raters did not agree. Given our results were reliant on a single rater per study, the report by Norman and co-workers [22] provided a valuable benchmark for inter-rater agreement across normal subjects and patients with sleep disordered breathing. The agreement between our algorithm and manual scoring for normal/mild and moderate severe SDB groups (Table 2) were equivalent to the inter-rater reliability reported by Norman for Stages N2 and N3. Our auto-scoring vs. manual agreement was similar to inter-rater reliability for stage Wake in normal/mild SDB. Our agreement was slightly inferior to the reported inter-rater reliability for REM and N1, which may be partially explained by the fact that our SDB patients were more severe than the Norman cohort (RDI = 41 ± 28 vs. 34 ± 31 events/hr). Suboptimal recognition of cortical arousals resulting from SDB may also have contributed to the difficulties in the algorithm staging REM sleep and differentiation between wake and stage N1. The reduced EMG bandwidth and slightly different electrode placement might also be responsible, as the accuracy of both REM and N1 detection was better in the original validation study on healthy controls in which the EMG power was derived from 40 to 128 Hz.

For this study we used as the differential inputs signals from the left and right EOG, rather than a standard placement used for development and initial validation of the algorithm. The only adaptation made was to accommodate differences in sampling rates. Thus, this study served to assess the generalizability of the staging algorithm using forehead EEG in patients with sleep disordered breathing, a chronic disease known to disrupt sleep architecture and compromise manual staging accuracy. We made no effort to manually edit the full-disclosure auto-scored epochs, although the capability is provided for physicians boarded in sleep medicine to review and interpret the staging. One of the advantages of staging sleep from the forehead is access to information provided by eye movements. Manually editing would definitely have eliminated obvious misclassifications between stage N1 and REM resulting from slow eye rolls apparent after long periods of wake. The disadvantage is that alpha waves useful in staging N1 are visually undetectable.

Others algorithms have been evaluated for the staging of sleep from the forehead. The Kappa coefficients obtained for wake, light non-REM (N1 and N2), N3 (SWS) and REM in the test group of 131 subjects using two EOG channels [25] were equivalent to the results we obtained in the moderate/severe SDB group. The inter-class correlations for time in N3 and REM across our SDB subjects were on average 20% better than results obtained in healthy subjects using a wireless forehead system [26]. The algorithms in this study was also equivalent to a five-state, single channel method based on sensor sites at Cz and Pz [26]. When comparing the accuracy of our automated scoring algorithm with the latter method, it is important to note that the sensitivity and positive predictive values presented in this study are based on one scorer per study, with multiple technicians used to score the data set. Conversely, Berthomier et al. [27] used two expert scorers and eliminated epochs in which raters disagree, an approach that can artificially inflated accuracy metrics by discarding epochs found difficult to score by human experts.

Because sleep staging is time consuming and somewhat subjective in nature, the auto-scoring can introduce a degree of consistency in the characterization of sleep architecture prior to visual inspection, a favorable approach when sleep EEG is used to evaluate outcomes such as those based on cognitive behavioral therapy [32,33]. Given the automated algorithm can be applied to stage sleep in real time and there were limited differences in RDI values calculated with total sleep times derived from manual vs. automated scoring from two electrooculographic leads, the algorithm could also be used to assist technicians identify two-hours of sleep time needed to transition from diagnosis to therapy in continuous positive airway pressure split night studies. Real time sleep staging could also be beneficial during assessment of sleep stage related RDI differences in patients undergoing an oral appliance titration study or in partial sleep-deprivation studies to allow the attending technician to monitor sleep stages of interest.

The combination of a lightweight, easily applied data acquisition system with automated sleep staging algorithms holds promise for routine application of sleep profiling in the evaluation of many disease states that are currently difficult to identify. As highlighted in the introduction, sleep disturbances are among the hallmark features of many psychiatric and neurological disorders and there is a need for objective biomarkers to confirm diagnosis, to identify sub-types and ultimately to ensure treatment efficacy. For example, periods of wake that serve as biomarkers of insomnia are distinct from the abnormally long and dense REM cycles for major depression. Differentiating depression from insomnia would be an important first step before introduction of a pharmacological or other intervention. In the case of depression, the normalization of the REM patterns, as seen in response to the Selective Serotonin Reuptake Inhibitors (SSRI), could be used as an indicator of treatment efficacy [34-36]. In contrast, sleep disturbances in PTSD and are often resistant to treatment with SSRIs [20,37]. Although there is substantial symptom overlap between depression and PTSD (e.g. depressed mood, anhedonia, social withdrawal, and decreased concentration), PTSD is characterized by disturbed sleep continuity with decreased total sleep time, poor sleep efficiency, minimal slow wave sleep and long but fragmented REM. More recently it has been suggested that fragmented REM sleep, characteristic of many patients with PTSD, may disrupt the process of encoding traumatic memories that normally allows for their recollection without the emotional charge and associated sympathetic arousal [20,38,39]. Drugs such as prazosin or clonidine, which act antagonistically at noradrenergic receptors, have recently been shown to improve sleep and relieve symptoms in some PTSD patients specifically reducing the number and severity of nightmares [40,41]. Thus, sleep profiling could be used to differentiate insomnia from depression or to indentify trauma victims at risk of developing PTSD and to improve the selection of therapy. Follow-up studies could evaluate the efficacy of prescribed pharmacological treatments in those diagnosed with psychiatric conditions.

## Conclusions

The level of agreement between automated sleep staging and visual scoring across all sleep stages other than N1 is approximately the same as the inter-rater agreement between scorers. Decreased auto-staging accuracy corresponds with increased sleep disordered breathing severity. These results suggest that the automated sleep staging algorithm may prove useful in evaluating sleep architecture patterns in patients with chronic diseases. Further investigation is required to determine if biomarkers derived from the automated staging will assist in the diagnosis and treatment of chronic diseases.

## Author contributions

Mr. Levendowski and Dr. Popovic performed the data analyses. All four authors contributed to the writing, editing and final approval of the manuscript.

## Disclosure statement

All authors are employees and shareholders of Advanced Brain Monitoring Inc. (Carlsbad, CA, USA), a company that develops and manufactures portable EEG recorders. All authors would benefit financially if the patent associated with the automated scoring algorithm was acquired by a third party.
